# Real-world treatment outcomes in multiple myeloma: Multicenter registry results from Finland 2009-2013

**DOI:** 10.1371/journal.pone.0208507

**Published:** 2018-12-05

**Authors:** Kari Remes, Pekka Anttila, Raija Silvennoinen, Mervi Putkonen, Hanna Ollikainen, Venla Terävä, Marjatta Sinisalo, Kristiina Kananen, Frida Schain, Päivi Castren-Kortegangas, Tiina M. Järvinen, Marta Pisini, Felix Wahl, Tricia Dixon, Amy Leval

**Affiliations:** 1 Turku University Hospital and University of Turku, Dept of Clinical Hematology, Turku, Finland; 2 University of Helsinki and Helsinki University Hospital Comprehensive Cancer Center, Department of Hematology, Helsinki, Finland; 3 Department of Medicine, Kuopio University Hospital, Kuopio, Finland; 4 Satakunta Central Hospital, Pori, Finland; 5 Päijät-Hämeen Central Hospital, Lahti, Finland; 6 Department of Internal Medicine, Tampere University Hospital, Tampere, Finland; 7 Kainuu Joint Authority for Social and Health Care, Clinic of Internal Medicine, Kajaani, Finland; 8 Janssen Cilag AB, Solna, Sweden; 9 Janssen Cilag OY, Espoo, Finland; 10 Janssen Cilag NV, Beerse, Belgium; 11 Department of Mathematics, Stockholm University, SE, Stockholm, Sweden; 12 JB Medical Ltd, The Old Brickworks, Sudbury, Suffolk, United Kingdom; 13 Department of Medical Epidemiology and Biostatistics, Karolinska Instutitet, Stockholm, Sweden; European Institute of Oncology, ITALY

## Abstract

Outcomes for patients with multiple myeloma (MM) have improved with the advent of novel therapies, however, real-world evidence of outcomes in clinical practice is scarce. We conducted a multi-center registry study to build a reliable picture of treatment and patient outcomes in Finland. The aim of this study was also to understand any methodological challenges in assessing treatment outcomes using disease registry data. Methods: We carried out a retrospective, observational study using data from the national Finnish Hematology Registry (FHR) to provide real-world evidence of outcomes for all adult patients diagnosed with and treated for MM between 2009–2013 at one of the six regional hospitals, with at least six months of recorded follow-up. Patients were identified within the FHR by applying eligibility criteria of a diagnosis of MM and verifiable records of medical treatment and lines of treatment during the study period. Patients receiving allogenic stem cell transplantation were excluded from the cohort, as were individuals who only had monoclonal gammopathy of undetermined significance diagnosis and patients who had not initiated treatment during this period. Kaplan Meier curves were used to calculate overall survival and time to next treatment. Stratification was carried out by drug status (conventional/novel) and by autologous stem cell transplant (ASCT) status. Results: A total of 321 patients met the inclusion criteria and were included in this study. Overall survival (OS) was longest in patients who received first-line novel therapy and ASCT (median not reached during 60-month follow-up) versus 46.2 months for novel first-line therapy without ASCT and 25.6 months for first-line conventional therapy without ASCT. Similarly, median time to next treatment were 33.9 months, 12.6 months and 7.8 months, respectively. Conclusions: The adoption of novel treatments in MM in Finland has had substantial impact on patient outcomes. Given the reality of complex treatment combinations for MM and relatively low patient numbers, assessing individual treatment effectiveness will require substantial cohort sizes and advanced, collaborative analytics on an international scale.

## Introduction

Multiple myeloma (MM) is an incurable hematological malignancy mainly affecting the elderly [1]. In patients with MM, neoplastic plasma cells accumulate in the bone marrow and produce a monoclonal protein (paraprotein) which is detected in the blood and/or urine and causes organ or tissue impairment [2].

Over recent years, the use of novel agents (immunomodulatory drugs [lenalidomide, thalidomide and pomalidomide] and the proteasome inhibitor [bortezomib]) and autologous stem cell transplant (ASCT) in patients eligible for transplant has improved the outcomes of patients with MM around the world [2–6]. However, MM remains incurable. Retrospective data from the Swedish National Registry reveals that survival increased dramatically in younger patients (<65 years) between 1950 and 2005 with the greatest improvements seen between the 1990s and the 2000s [7] and that survival continues to improve with the increased use of novel agents in all age groups [5, 8].

European Society of Medical Oncology (ESMO) guidelines are regularly updated [9–15] and have consistently considered clinical condition (fitness) and age to be critical in deciding on treatment options. Patients aged <65 years or fit patients <70 years in good clinical condition are eligible for ASCT and novel chemotherapy, patients aged >65 years or patients ineligible for ASCT receive novel therapy if fit enough, or conventional chemotherapy if not. Novel therapies were first mentioned in the 2007 guidance [10]. Similarly, the treatment algorithm in Finland recommends treatment according to age. First-line treatment for younger patients is induction combination chemotherapy using novel agents, followed by ASCT. For older patients chemotherapy plus novel agents, if tolerated, is recommended without ASCT [6]. More recent treatment recommendations from Finland (2017) have raised the age threshold from 70 years; patients aged 70–75 years are eligible for transplant if fit enough, transplant ineligible patients aged up to 85 years who are fit enough for novel agents are treated with novel chemotherapy and unfit patients and those aged over 85 years are treated with conventional [16].

There are numerous first-line treatment options, regardless of whether patients are eligible for ASCT or not. Complexity emerges in the choice of further lines of treatment and sequencing of treatments. Treatment second-line and beyond includes the novel agents in various combinations. Treatment choice varies according to patient-specific characteristics, previous treatments (type, efficacy and tolerance), number of prior treatment lines, remaining treatment options and interval from the last therapy.

As new treatments become a part of clinical practice, the outcomes must be robustly evaluated outside the clinical trial setting to ensure that patient care is improved compared with existing treatments. Retrospective data collections are invaluable in assessing real-life outcomes. Cancer registries often rely on retrospective data collection, utilizing patient medical records to collect the key variables for clinical outcomes. Many cancer registries exist, especially in the Nordic countries, with detailed information captured at diagnosis and cause of death [17]. However, treatments and outcomes between diagnosis and death are rarely captured. As new therapies develop and require real-life comparisons, understanding the entire patient care trajectory is critical in establishing new treatment strategies and their timely reimbursement, if appropriate.

### Study objectives

The objectives of this study are to investigate real-world MM treatment outcomes in Finland and to understand any methodological challenges in using retrospective registry data in assessing these specific outcomes.

## Methods

The study design was non-interventional and retrospective. Available data were extracted from the Finnish Hematology Registry (FHR); patient medical records from six regional hospitals were used to fill and validate FHR data gaps. The FHR was established in 2009 where all diagnosed patients are asked to give their consent to be included, and thus the first patient consents were available from that year. Any data prior to late 2009 was thus inserted retrospectively, after the consent had been received. The FHR did not include any deceased patient data in the register and thus all the patients diagnosed prior to 2009 had to survive until late 2009 at least to be able to give a consent. As such, for the time-period prior to 2009, the FHR contained a selection bias due to excluded immortal time for many hospitals´ data entry, and, accordingly, the cohort entry date in this study was limited to those patients diagnosed from 2009 onward. Patients were identified within the FHR by applying eligibility criteria of a diagnosis of MM and verifiable records of medical treatment and lines of treatment during the study period. Patients’ data were screened for eligibility according to diagnosis, time period, age, medical treatment records and treatment line records.

Inclusion criteria were as follows: aged over 18 years, diagnosis of MM between 2009 and 2013 and at least 6 months of follow-up according to the protocol. Exclusion criteria for analysis were as follows: allogeneic SCT, monoclonal gammopathy of undetermined significance and patients who did not receive drug treatment.

Patients gave their informed consent before any patient data were entered into the FHR. The consent mentions that data from the registry may be used for collaborative projects with national study groups and commercial organisations. The FHR’s steering committee reviews research proposals on FHR data and the ethics committee for this project was Coordinating Ethics Committee of the Helsinki and Uusimaa Hospital Region. The committee approved this study, with reference number was 68/13/03/00/2013.

Data collected included demographic data at the time of diagnosis, details of treatment (drug and ASCT) and treatment line, outcomes and date of last follow-up if the patient was still living.

Novel agents were defined as lenalidomide, thalidomide, bortezomib and pomalidomide. Line of treatment was defined in the protocol as one or more cycles of a planned treatment, e.g. induction followed by ASCT would count as one line of treatment and a new line of treatment was defined as modification of planned course of therapy to include other treatment agents (alone or in combination) as a result of disease progression, relapse, or toxicity.

Descriptive statistics and Kaplan Meier curves were used to calculate time to next treatment (TTNT) and overall survival (OS) in months for first-line and second-line treatment. Stratification was carried out by drug status (conventional/novel) and by ASCT status.

## Results

The study was carried out at six sites in Finland (Helsinki University Hospital, Turku University Hospital, Kuopio University Hospital, Tampere University Hospital, Satakunta Central Hospital, Kainuu Central Hospital) where the majority of patients with newly diagnosed MM are treated in Finland. Data from 321 patients diagnosed and treated between 2009–2013 and who met the study inclusion criteria were analyzed. It should be noted that although the FHR is a national, population-based quality, treatment and research registry, input is not fully comprehensive and verifiable at all hospitals, since there are around 380 new MM cases annually in Finland including smoldering MM [18]. Median follow-up time (i.e. person time) for included patients was 25 months, with a maximum follow-up of 60 months.

### Demographics at diagnosis

The demographics at diagnosis are shown in Table 1, stratified by first-line treatment choice.

**Table 1:** Demographic details of patients included in the study.

	Non-ASCT			ASCT	Total
Treatment at first line	Novel	Conventional		Novel + ASCT	
**Patients, n**	161	46		114	321
**Male, n (%)**	72 (45%)	21 (46%)		45 (51%)	138 (43%)
**Median age at diagnosis, years (range)**	69 (45–83)	77.5 (47–89)		61 (42–70)	66 (42–89)
**Age distribution, n (%)**					
<40 years	0 (0%)		0 (0%)	0 (0%)	0 (0%)
40–49 years	3 (2%)		2 (4%)	4 (4%)	9 (3%)
50–59 years	18 (11%)		0 (0%)	36 (32%)	54 (17%)
60–64 years	29 (18%)		2 (4%)	47 (41%)	78 (24%)
65–69 years	32 (20%)		4 (9%)	26 (23%)	62 (19%)
70–74 years	41 (25%)		9 (20%)	1 (1%)	51 (16%)
≥ 75 years	38 (24%)		29 (63%)	0 (0%)	67 (21%)
**Type of MM, n (%)**					
IgA	21 (13%)		6 (13%)	15 (13%)	42 (13%)
IgD	0 (0%)		1 (2%)	1 (1%)	2 (1%)
IgG	58 (36%)		15 (33%)	53 (46%)	126 (39%)
Bence Jones	37 (23%)		9 (19%)	27 (24%)	73 (23%)
Missing	45 (28%)		15 (33%)	18 (16%)	78(24%)
**Osteolytic bone lesions, n (%)**					
0	48 (30%)		15 (33%)	26 (23%)	89 (28%)
1	18 (11%)		3 (6%)	10 (9%)	31 (10%)
>1	85 (53%)		23 (50%)	74 (65%)	182 (57%)
Missing	10 (6%)		5 (11%)	4 (3%)	19 (6%)
**Laboratory values at diagnosis, median**					
Creatinine, μmol/l	87 (2% missing)		86 (0% missing)	79 (3% missing)	84 (2% missing)
Beta-2 microglobulin (β2m), mg/l	3.25 (35% missing)		4.45 (57% missing)	2.9 (28% missing)	3.2 (36% missing)
Hemoglobin (Hb), g/l	107 (0% missing)		103 (0% missing)	110.5 (2% missing)	108 (1% missing)
**Durie-Salmon, n (%)**					
I	13 (8%)		3 (7%)	13 (11%)	29 (9%)
II	21 (13%)		5 (11%)	24 (21%)	50 (16%)
III	22 (14%)		7 (15%)	31 (27%)	60 (19%)
Missing	105 (65%)		31 (67%)	46 (40%)	182 (57%)

### First-line treatment choice

**Fig 1:**
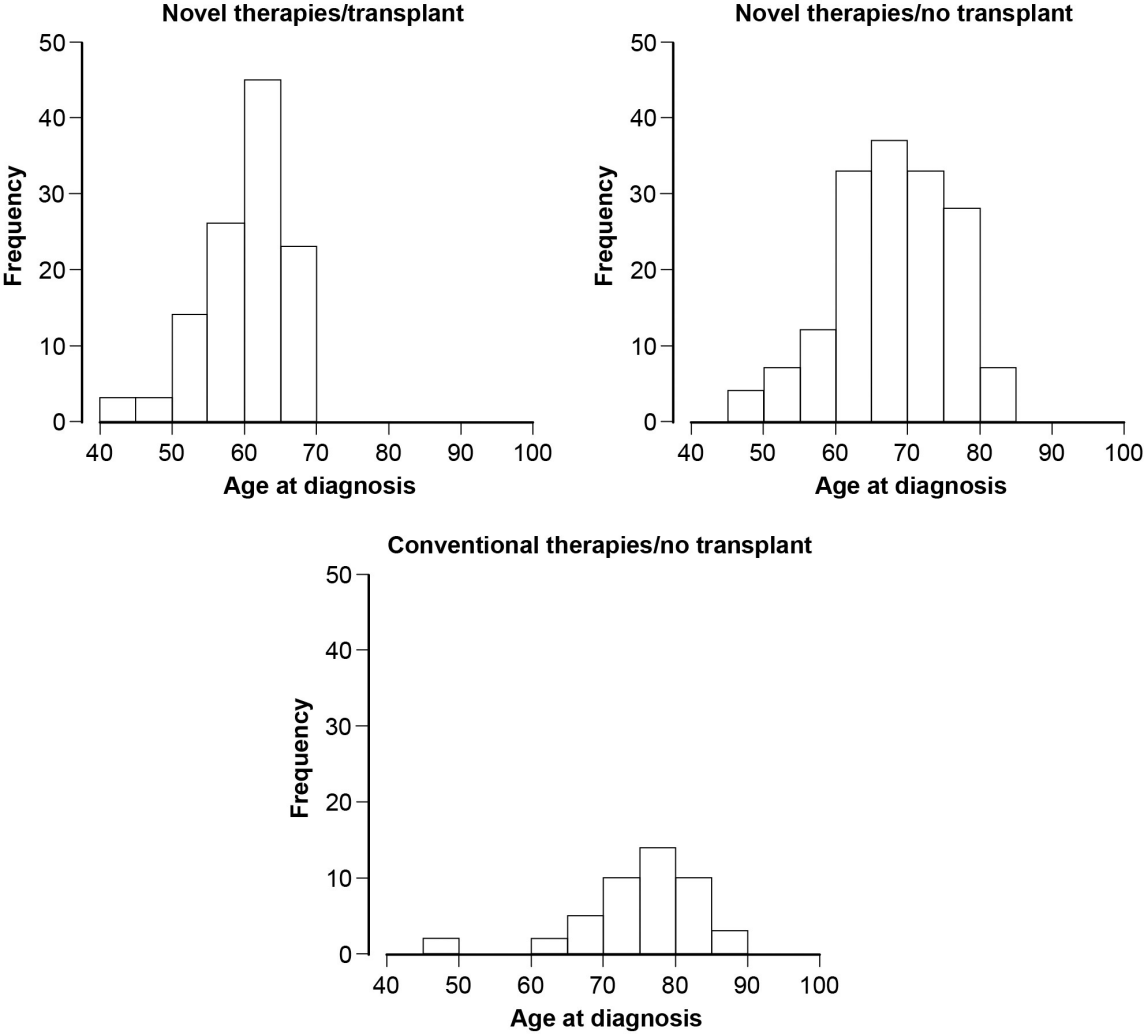
First-line treatment choice by age at diagnosis.

Overall, almost 90% of patients received novel therapies (275/321, 86%). In accordance with the Finnish advice at the time, age was the key determinant of treatment (novel or conventional agents, ASCT or not, Fig 1).

### Outcomes

**Fig 2:**
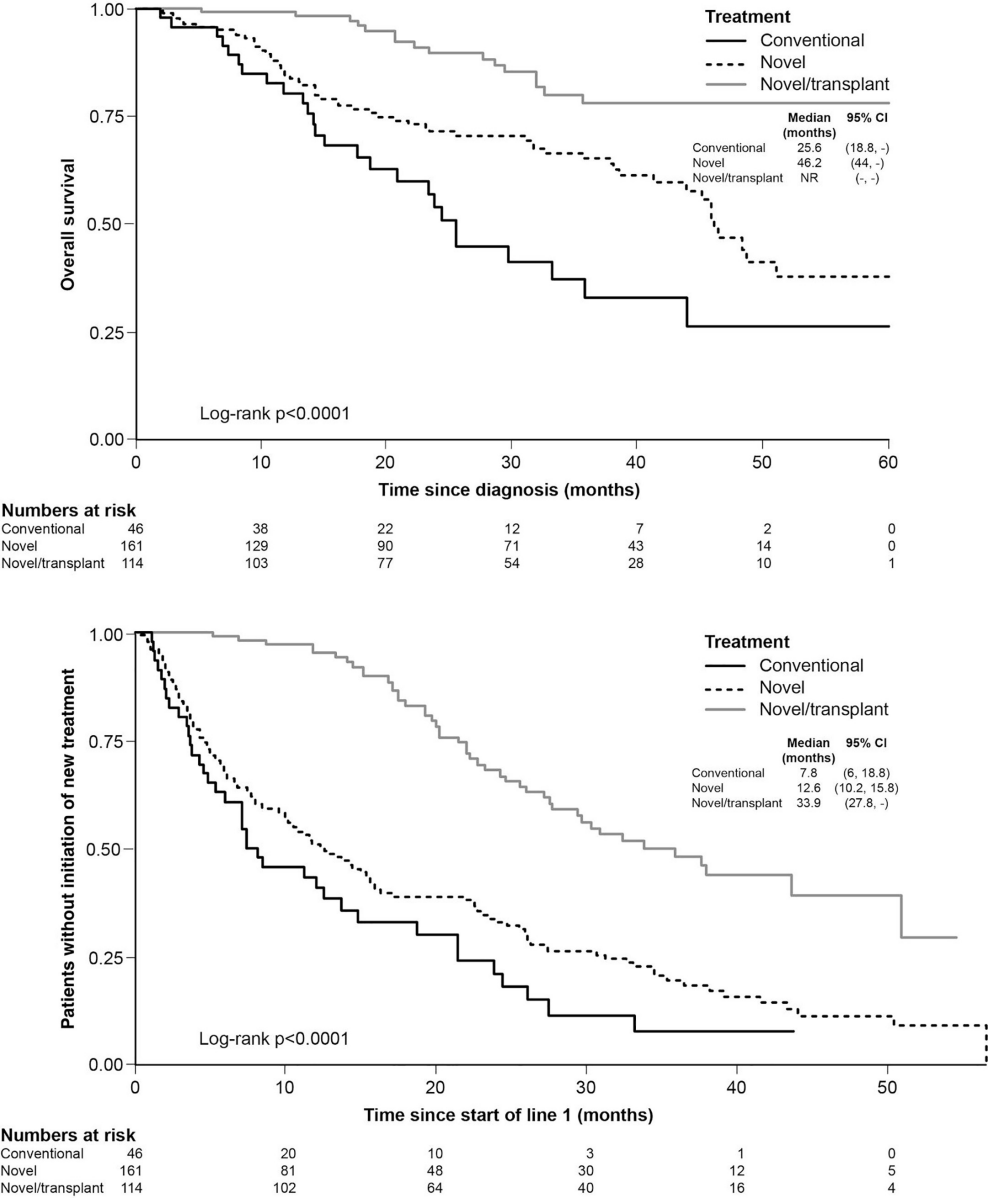
OS (top) and TTNT (bottom) by first-line treatment choice (transplant and non-transplant patients).

**Fig 3:**
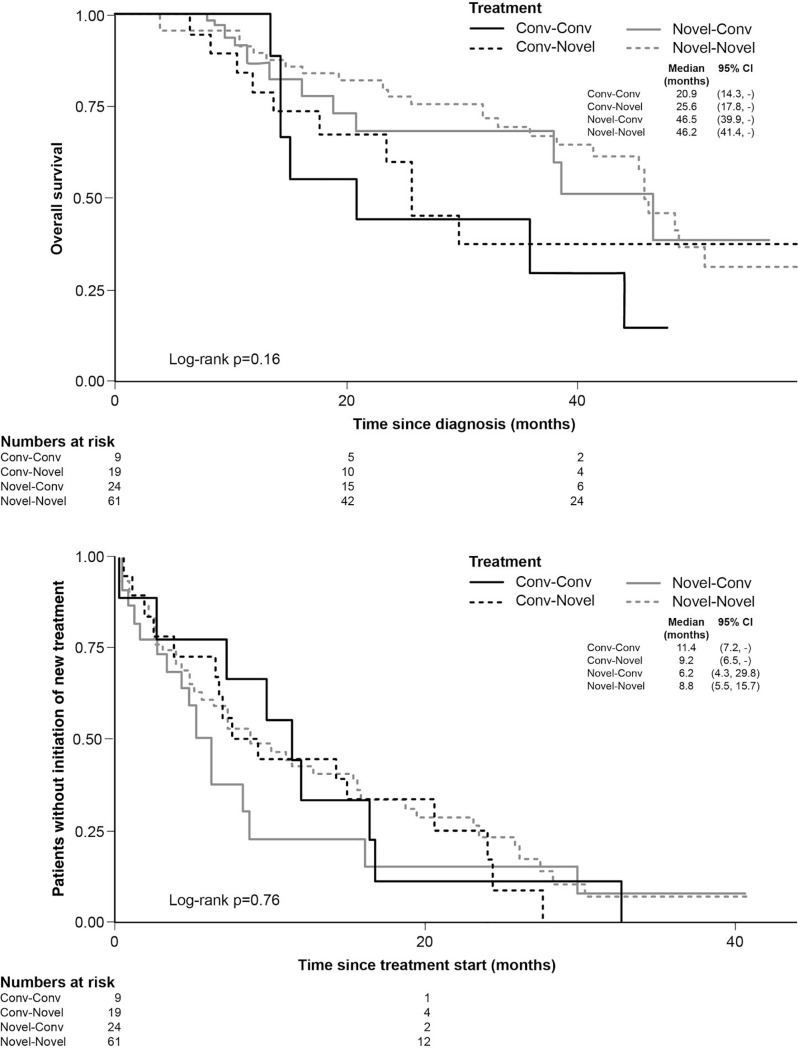
OS (top) and TTNT (bottom) by first-and second-line treatment choice (non-transplant patients only).

Patients with transplant and novel agents in first-line treatment had the best outcomes (OS and TTNT), followed by novel agents alone and finally conventional agents alone (Fig 2). Limited data was available for non-transplanted patients who had received two lines of treatment. For non-transplanted patients, there was a trend towards extended OS in patients who received novel agents in first line and novel or conventional agents in second line (median OS 46.2 months for novel followed by novel and 46.5 months for novel followed by conventional), versus those who received conventional agents in first line and novel agents in second line (median OS 25.6 months) or conventional agents in both treatment lines (median OS 20.6 months), illustrating the importance of early treatment with novel agents (Fig 3, top). No significant differences in TTNT in second-line were observed between the different treatment groups (Fig 3, bottom). It should be noted that patient numbers are too low for meaningful comparisons.

## Discussion

This study shows that clinical practice in Finland between 2009 and 2013 broadly reflected guidelines [6, 14], with the majority of younger patients receiving ASCT and novel therapies first-line and older patients receiving chemotherapy alone. Overall, most patients received novel agents as first-line treatment (86%). Similar results were seen in a retrospective US-based study which used data from the National Cancer Institute Surveillance, Epidemiology and End Results cancer registries. Of the patients diagnosed with MM in 2007 (n = 742) in the US study, 75.5% received a novel agent within 12 months of diagnosis [19].

The treatment choices were reflected as poorer outcomes for older patients receiving conventional therapy in Finland between 2009 and 2013. However, data from several recent studies have shown that use of novel agents in older people improves OS. Retrospective data from the national Swedish registry (diagnosed with MM between 2000 and 2011) revealed that in the elderly the use of novel agents first-line without ASCT resulted in a significantly longer median OS versus conventional therapies (4.9 years versus 2.3 years, p<0.0001) [5]. A retrospective study carried out in the US reported the outcomes of 117 elderly MM patients aged over 70 years who did not undergo ASCT [20]. A considerable proportion of these patients (83%) received novel agents which resulted in improved outcomes; patients receiving a two-drug regimen had median OS of 7.1 years and median OS was not reached in those receiving three- or four-drug regimens.

The results from the present national registry study revealed that the best outcomes were seen, as expected, with novel agents plus ASCT as first-line treatment. We provide real-world evidence that early use of novel therapy improves outcomes, confirming the findings of other retrospective studies from the Nordics [8, 21] and elsewhere [22, 23]. Our real-world OS results are comparable to those reported in many studies in young and elderly MM cohorts [24], although longer OS have been observed in selected phase 3 trials [25].

The outcome results from the FHR data presented here are hampered by challenges and pitfalls in collecting and analyzing the available retrospective registry data. Lack of complete patient records made it impossible to extract treatment outcomes from the 1990s to the early 2000s from the FHR. Accordingly, exclusion of this period leads to a substantial underestimation of the impact of novel treatment options. However, it is clear that outcomes have improved over time; a study published in 1999 showed that MM patients under 70 years in Finland and treated with chemotherapy only had a median OS of 49 months [26]. Shifts in standard treatment regimens over time make comparing outcomes between time periods difficult, but median OS in the present study was not reached in the population who received ASCT and novel therapies, with a follow-up of 60 months.

Although the Nordic countries are well known for their solid tumor registries, registries in hematology are less well-established and there is a paucity of real-world retrospective studies looking at treatment patterns in patients with MM over time in the Nordics. Several large studies from the US provide real-world evidence of variation in treatment options and in treatment sequencing, as seen in the present study. They show that bortezomib is the mainstay of treatment from first to third-line treatment, with second-generation novel agents (carfilzomib and pomalidomide) used as later lines of therapy since 2013. Numerous treatment options are used in first-line and beyond, with varying treatment sequences [27–29].

Real-world evidence is important for evaluating on-going practice, including the use of new agents in different treatment lines, and is crucial to facilitate patient access to new treatments, developing criteria for stopping one treatment and starting the next, understanding treatment sequencing and informing clinical trial design. There is a paucity of adequate real-world data, which could be utilized by national and local authorities in making decisions on treatment access.

However, there are limitations and methodological challenges in using registries to provide real-world evidence in hematological cancers. The key limitation is the quality of data collection and the heterogeneity of data available to collect from everyday clinical practice. When collecting real-world data, it is essential that key source data is standardized within patient records and can be easily transferred to the registry and collected within a normal clinical practice setting. At present standardization is not commonplace and many key outcome variables are currently retrieved from free-text fields. Standardized data collection systems would also ensure that users input all the essential data, including cytogenetics and risk grouping, which would minimize missing data within the registry. In the present study, bias in treatment selection and definition of treatment lines, missing data such as cytogenetic data and low patient numbers in further treatment lines due to deficiencies in the follow-up data made accurate stratified multivariate adjusted models impossible to generate. Also, not automating and requiring data entry in to a registry leads to a potential patient selection bias. In 2013 for example, 54 MM patients from the Helsinki centers were entered into the FHR while the mandatory Finnish Cancer Registry (with information on diagnosis but neither treatment nor outcomes) reported 114 incident MM cases from the entire Helsinki region. Smaller clinics, treating older MM patients who do not receive ASCT, are less likely to enter data into the FHR according to national experts. This bias could lead to an overestimation of the OS among MM patients in the current study. Table 2 lists potential solutions to the challenges faced in using real-world retrospective registry data in this disease area.

**Table 2:** Challenges in using a retrospective study design to understand real-world experience and potential solutions.

Issue	Solution
Cancer registries traditionally set up for solid tumors	Design cancer registries specifically for hematological tumors
Data quality heterogeneous–incomplete or relevant data not collected	Predetermined outcome parameters defined Structured patient data from electronic journals to enable direct derivation
Potential patient selection bias	Automated data extractions based upon diagnostic criteria and permissions granted
Lack of accurate progression data	TTNT potentially more reliable
Low patient numbers, especially in later treatment lines	Share data across countries International registry Common data model
Complex treatment pathway	Predetermined outcome parameters defined Use clinical support decision tools to record practice prospectively

Treatment line definition differs in real-life clinical practice, with some clinicians adding on new treatments to deepen response for example, while still considering it part of the current treatment line, while another clinician, or the retrospective record, might classify that as a new treatment line. Although treatment lines were defined in the protocol, it was up to practicing clinicians to document line of treatment as per their standard practice, introducing variations and potential uncertainty.

Similar data limitations have been observed in a recent Dutch study which compared data from a bortezomib clinical trial in MM to real-world data. The real-world data was limited by missing data in patients’ records, lack of information on prognostic factors e.g. biomarkers or measures of frailty and variation in treatment patterns [30].

Outcome data from this study focuses on first and second-line treatments; data from multiple lines of therapy was limited by small patient numbers and is excluded from the analysis. We believe that it would be more valuable to be able to follow the whole patient journey. Clinical decision support tools are available and are used in the Nordics in other disease areas, for example prostate cancer [31] and human immunodeficiency virus [32], and a similar approach for MM would be helpful. Such tools not only allow longitudinal treatments and patient responses to be collected and displayed, but also make comprehensive patient overview easier for the treating physician and their team. Importantly for real-world studies, these tools drive the standardization of variables in clinical practice.

Progression free survival (PFS) is the usual measure of progression in clinical trials, but is not considered an outcome in clinical practice. PFS is very difficult to determine accurately from patient records, since it is generally confirmed by monitoring, and a patient may have progressed prior to scans being carried out. In MM specifically, some agents are not given until progression, additionally, some clinicians do not treat until progression in order to avoid treatment resistance or to reduce costs. In our study, progression data was missing from many clinical records. Therefore, we considered that TTNT might be a more objective measure [3]. However, TTNT is not without limitations and does not always accurately reflect treatment effectiveness since the reasons for starting a new therapy are not always related to disease progression and may vary between different centers. Indeed, in the present study, TTNT seemed to be shorter for non-transplanted patients receiving novel treatments in both first and second line than those receiving only conventional treatment in both first and second-line, although patient numbers were small. This is possibly due to these patients having more aggressive or fast progressing disease, though it also may indicate a change in clinical practice in that novel treatments are started earlier than conventional treatments, which clinicians generally initiate once end-organ damage has been incurred.

Collaboration across countries would be valuable to increase patient numbers needed for more robust analyses; however, it will be important to ensure data quality across countries and that differences in treatment guidelines are understood. Europe’s Innovative Medicines Initiative (IMI) project HARMONY (Healthcare alliance for resourceful medicines offensive against neoplasms in hematology) will do just this with its pan-European stakeholder network, creating the largest ever synchronized data source for hematology outcomes [33]. HARMONY will use real-life data to define relevant clinical endpoints and disease outcomes applicable to clinicians and policy makers, increasing the understanding of treatment outcomes in MM, while supporting the optimal treatment choices provided to patients over the course of their disease.

## Conclusion

Registry data shows that the adoption of novel treatments in MM has had substantial impact on patient outcomes and that age is the key determinant of treatment choice and OS. In Finland between 2009 and 2013, most younger (<70 years) patients received ASCT and novel therapies as first-line treatment and had far superior OS and TTNT versus those patients who did not receive this combination treatment. Of patients who did not receive ASCT, those who received novel therapies had superior outcomes to those who received conventional therapies alone. Since 2013, MM treatment has progressed substantially with additional novel agents currently in use.

In future, given the reality of complex treatment combinations and sequencing, together with relatively low patient numbers, assessing individual treatment effects will require substantial cohorts and advanced, collaborative analytics on an international scale, such as those planned for hematology in the IMI HARMONY project. Knowledge of real-life treatment outcomes can facilitate patient access to novel treatments and should be used to inform and improve trial design. Trial endpoints should be feasible to assess in clinical practice settings, so that proper real-world comparisons can be made. There is a clear need for real-world data to shed light on everyday practice and outcomes outside of clinical trials.

## Abbreviations

ASCT: Autologous stem cell transplant
ESMO: European Society of Medical Oncology
FHR: Finnish Hematology Registry
HARMONY: Healthcare alliance for resourceful medicines offensive against neoplasms in hematology)
IMI: Innovative Medicines Initiative
MM: Multiple myeloma
OS: Overall survival
PFS: Progression free survival
TTNT: Time to next treatment
TTP: Time to progression
